# Supplementary material for: Multi-Omics Analysis of SOX4, SOX11, and SOX12 Expression and the Associated Pathways in Human Cancers
Source: J Pers Med. 2021 Aug 23;11(8):823. doi: 10.3390/jpm11080823 (PMC8400412; doi:10.3390/jpm11080823)
Supplement: Supplementary file 1 [file jpm-11-00823-s001.zip › jpm-1272270-supplementary.pdf]

Supplementary information: Multi-omics Analysis of SOX4, SOX11, and SOX12 Expression and the Associated Pathways in Human Cancers

Jaekwon Seok [1,†], Minchan Gil [1,†], Ahmed Abdal Dayem [1,] Subbroto Kumar Saha [1] and Ssang-Goo Cho [1,*]

[1] Department of Stem Cell and Regenerative Biotechnology, Molecular & Cellular Reprogramming Center (MCRC), Incurable Disease Animal Model & Stem Cell Institute (IDASI), Konkuk University, 120 Neungdong-ro, Gwangjin-gu, Seoul 05029, Korea; tjrwornjs@naver.com (J.S.); minchangil@gmail.com (M.G.); ahmed_morsy86@yahoo.com (A.A.D.); subbroto@konkuk.ac.kr (S.K.S.);

* Correspondence: ssangoo@konkuk.ac.kr; Tel.: +82-2-450-4207 or +82-2-444-4207

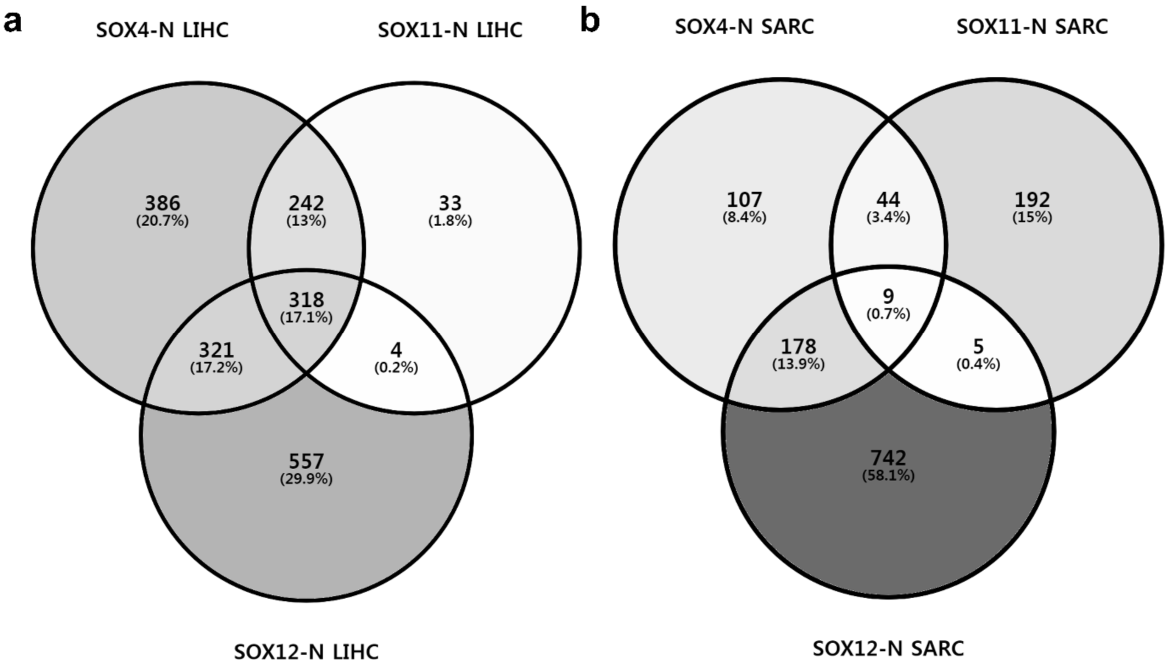

Supplementary Figure S1. Analysis of negatively correlated genes of SOX4, SOX11, and SOX12 (a) Venn diagram of negatively correlated genes with three SOX genes using TCGA-LIHC transcriptome datasets from R2 database. (b) Venn diagram of negatively correlated genes with three SOX genes using TCGA-SARC transcriptome datasets from R2 database.

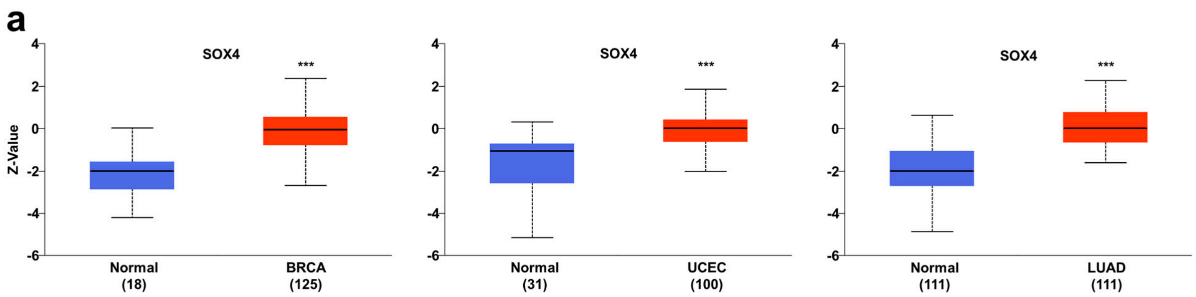

Supplementary Figure S2. Protein expression profile of SOX4 in various types of cancers and their adjacent normal tissues. (a) Box plot of protein expression level of SOX4 in BRCA, UCEC, and LUAD (plotted in red) and adjacent normal tissues (plotted in blue). (***: p<0.0001). BRCA: breast carcinoma; UCEC: uterine corpus endometrial carcinoma.

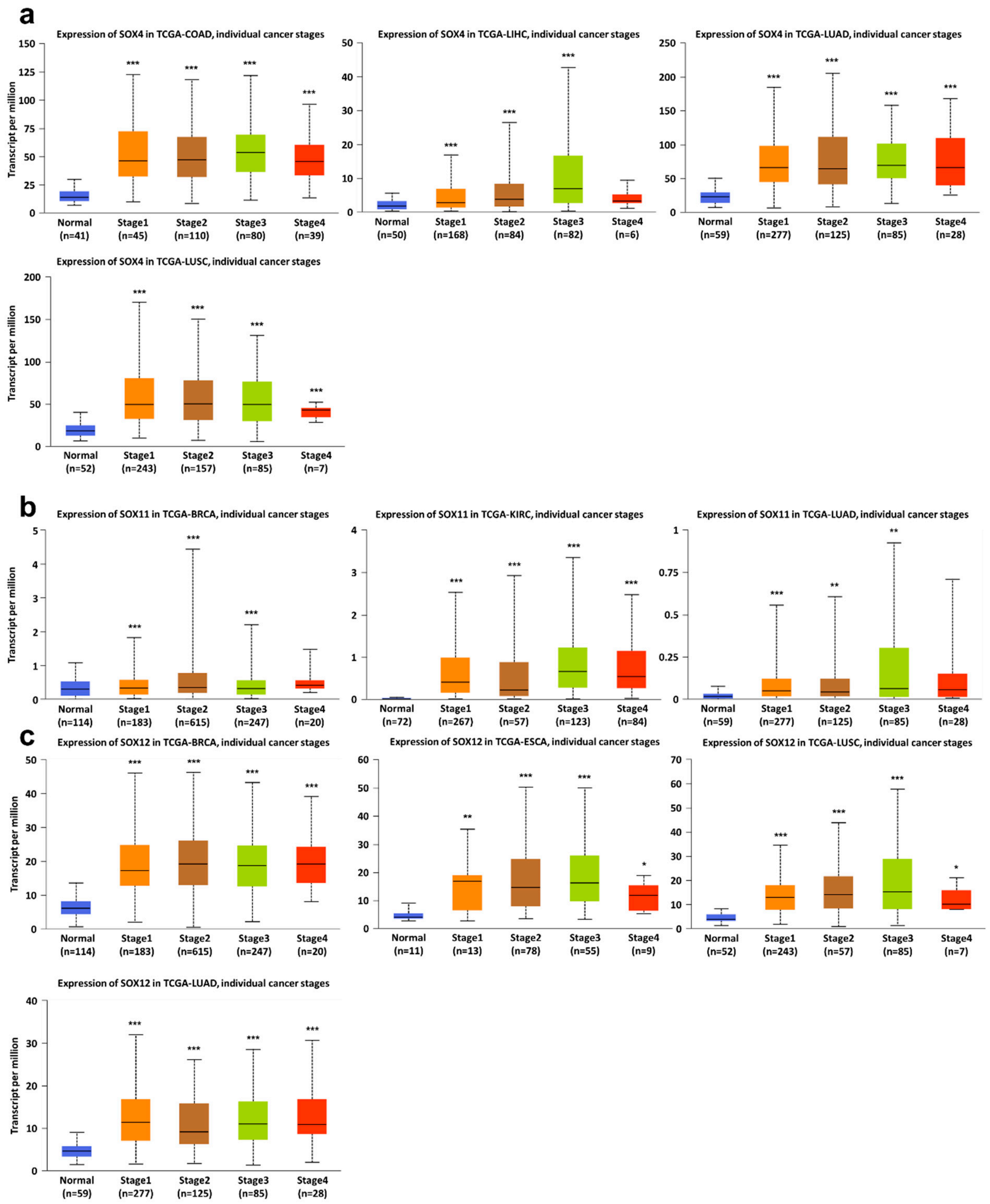

Supplementary Figure S3. mRNA expression level of SOX4, SOX11, and SOX12 in multiple cancer types. (a) expression level of SOX4 in COAD, LIHC, LUAD, and LUSC according to the stage of cancer compared to adjacent normal tissues. (b) expression level of SOX11 in BRCA, KIRC, and LUAD according to the stage of cancer compared to adjacent normal tissues. (c) expression level of SOX12 in BRCA, ESCA, LUAD, and LUSC according to the stage of cancer compared to adjacent normal tissues (***:** $p < 0.05$; **: $p < 0.01$; ***: $p < 0.001$).

a

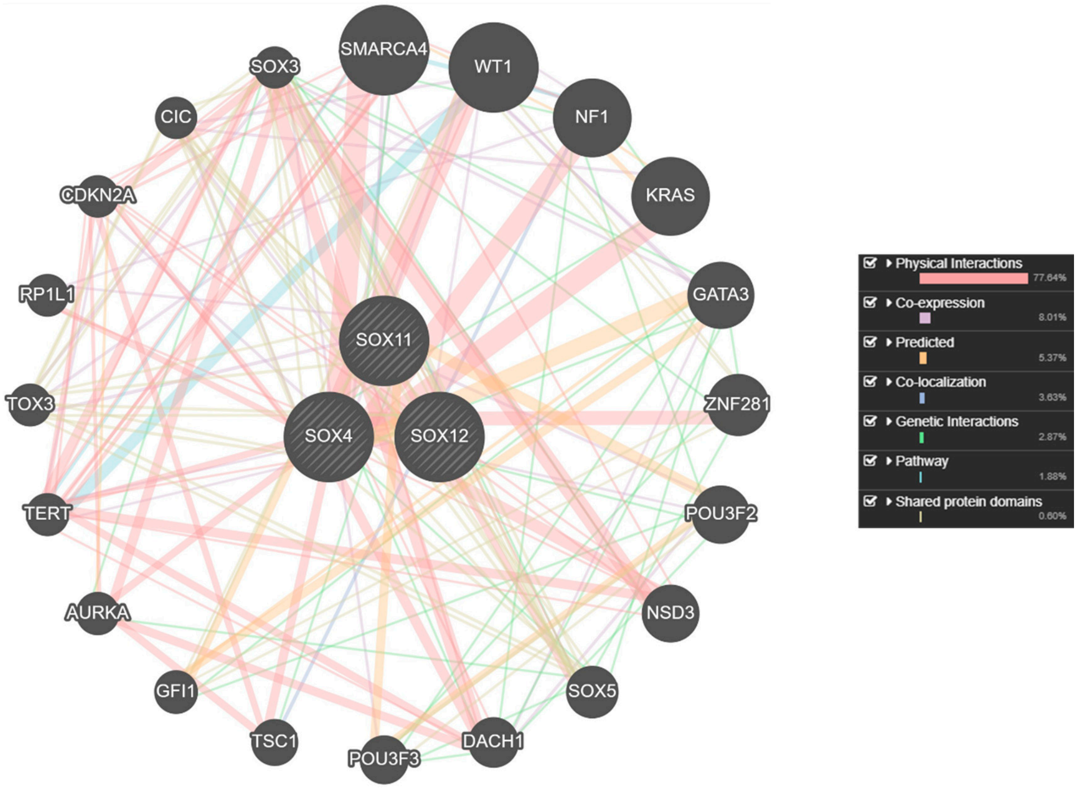

Supplementary Figure S4. gene network analysis with SOX4, SOX11, and SOX12 using GeneMANIA database. (a) Line thickness indicates the intensity of interaction. The line color demonstrates the interaction categories including physical interactions, co-expression, predicted, co-localization, genetic interactions, pathway, and shared protein domains.

Supplementary Table S1. The Reactome pathway analysis of negatively correlated genes with three SOX genes using LIHC-TCGA transcriptome datasets.

Pathway identifier	Pathway name	Entities p-Value
R-HSA-1430728	Metabolism	1.11E-16
R-HSA-9033241	Peroxisomal protein import	4.02E-14
R-HSA-211859	Biological oxidations	1.08E-12
R-HSA-211945	Phase I - Functionalization of compounds	2.70E-11
R-HSA-9609507	Protein localization	4.31E-11
R-HSA-8978868	Fatty acid metabolism	1.18E-09
R-HSA-556833	Metabolism of lipids	5.67E-09
R-HSA-71291	Metabolism of amino acids and derivatives	6.00E-09
R-HSA-211979	Eicosanoids	4.14E-07
R-HSA-211935	Fatty acids	1.24E-06
R-HSA-211897	Cytochrome P450 - arranged by substrate type	2.53E-06
R-HSA-390918	Peroxisomal lipid metabolism	5.80E-06
R-HSA-71384	Ethanol oxidation	1.08E-05
R-HSA-1989781	PPARA activates gene expression	1.80E-05
R-HSA-400206	Regulation of lipid metabolism by PPARalpha	2.06E-05
R-HSA-389661	Glyoxylate metabolism and glycine degradation	2.46E-05
R-HSA-177128	Conjugation of salicylate with glycine	7.15E-05
R-HSA-9657689	Defective SERPING1 causes hereditary angioedema	7.38E-05
R-HSA-70895	Branched-chain amino acid catabolism	1.66E-04
R-HSA-193368	Synthesis of bile acids and bile salts via 7alpha-hydroxycholesterol	2.03E-04
R-HSA-140837	Intrinsic Pathway of Fibrin Clot Formation	2.58E-04
R-HSA-194068	Bile acid and bile salt metabolism	3.97E-04
R-HSA-156587	Amino Acid conjugation	4.59E-04
R-HSA-159424	Conjugation of carboxylic acids	4.59E-04
R-HSA-390247	Beta-oxidation of very long chain fatty acids	5.40E-04
R-HSA-5365859	RA biosynthesis pathway	6.17E-04
R-HSA-140877	Formation of Fibrin Clot (Clotting Cascade)	6.74E-04
R-HSA-9614085	FOXO-mediated transcription	7.21E-04
R-HSA-211958	Miscellaneous substrates	7.76E-04
R-HSA-9033500	TYSND1 cleaves peroxisomal proteins	0.001270915
R-HSA-2142753	Arachidonic acid metabolism	0.001579895
R-HSA-9024446	NR1H2 and NR1H3-mediated signaling	0.001940267
R-HSA-9615017	FOXO-mediated transcription of oxidative stress, metabolic and neuronal genes	0.001958166

R-HSA-2142816	Synthesis of (16-20)-hydroxyeicosatetraenoic acids (HETE)	0.001984323
R-HSA-8963684	Tyrosine catabolism	0.001984323
R-HSA-9651496	Defects of contact activation system (CAS) and kallikrein/kinin system (KKS)	0.002363724
R-HSA-9671793	Diseases of hemostasis	0.002363724
R-HSA-2161517	Abacavir transmembrane transport	0.002389212
R-HSA-3371599	Defective HLCS causes multiple carboxylase deficiency	0.002389212
R-HSA-5579029	Metabolic disorders of biological oxidation enzymes	0.002396801
R-HSA-2142691	Synthesis of Leukotrienes (LT) and Eoxins (EX)	0.00262302
R-HSA-192105	Synthesis of bile acids and bile salts	0.002811605
R-HSA-432030	Transport of glycerol from adipocytes to the liver by Aquaporins	0.002874212
R-HSA-159418	Recycling of bile acids and salts	0.003040886
R-HSA-5362517	Signaling by Retinoic Acid	0.003066134
R-HSA-156580	Phase II - Conjugation of compounds	0.003555906
R-HSA-1614635	Sulfur amino acid metabolism	0.003559625
R-HSA-77111	Synthesis of Ketone Bodies	0.003795718
R-HSA-3323169	Defects in biotin (Btn) metabolism	0.003974202
R-HSA-76005	Response to elevated platelet cytosolic Ca2+	0.004965026
R-HSA-9657688	Defective factor XII causes hereditary angioedema	0.005023115
R-HSA-8957322	Metabolism of steroids	0.005145457
R-HSA-8963691	Phenylalanine and tyrosine metabolism	0.005689156
R-HSA-71032	Propionyl-CoA catabolism	0.00607555
R-HSA-177135	Conjugation of benzoate with glycine	0.00607555
R-HSA-74182	Ketone body metabolism	0.006488823
R-HSA-77289	Mitochondrial Fatty Acid Beta-Oxidation	0.006776481
R-HSA-1369062	ABC transporters in lipid homeostasis	0.007318421
R-HSA-9018682	Biosynthesis of maresins	0.007318421
R-HSA-9029569	NR1H3 & NR1H2 regulate gene expression linked to cholesterol transport and efflux	0.008137183
R-HSA-166658	Complement cascade	0.008456402
R-HSA-389887	Beta-oxidation of pristanoyl-CoA	0.00918079
R-HSA-114608	Platelet degranulation	0.01050252
R-HSA-8964540	Alanine metabolism	0.010923388
R-HSA-5679090	Defective ABCG8 causes gallbladder disease 4 and sitosterolemia	0.010923388
R-HSA-5679096	Defective ABCG5 causes sitosterolemia	0.010923388
R-HSA-5668914	Diseases of metabolism	0.01166481
R-HSA-2161522	Abacavir transport and metabolism	0.012511894

R-HSA-196780	Biotin transport and metabolism	0.013816394
R-HSA-8963899	Plasma lipoprotein remodeling	0.014149649
R-HSA-211981	Xenobiotics	0.014481606
R-HSA-174824	Plasma lipoprotein assembly, remodeling, and clearance	0.01512893
R-HSA-382556	ABC-family proteins mediated transport	0.015528391
R-HSA-196849	Metabolism of water-soluble vitamins and cofactors	0.017387467
R-HSA-5661270	Formation of xylulose-5-phosphate	0.017965672
R-HSA-6798163	Choline catabolism	0.017965672
R-HSA-9027307	Biosynthesis of maresin-like SPMs	0.017965672
R-HSA-2408508	Metabolism of ingested SeMet, Sec, MeSec into H2Se	0.019609123
R-HSA-400253	Circadian Clock	0.02016351
R-HSA-2142670	Synthesis of epoxy (EET) and dihydroxyeicosatrienoic acids (DHET)	0.022733501
R-HSA-5579026	Defective CYP11A1 causes Adrenal insufficiency, congenital, with 46,XY sex reversal (AICSR)	0.023359471
R-HSA-196854	Metabolism of vitamins and cofactors	0.024212916
R-HSA-3296482	Defects in vitamin and cofactor metabolism	0.024852799
R-HSA-1368108	BMAL1:CLOCK,NPAS2 activates circadian gene expression	0.024852799
R-HSA-9616334	Defective Base Excision Repair Associated with NEIL1	0.025632703
R-HSA-193775	Synthesis of bile acids and bile salts via 24-hydroxycholesterol	0.026770851
R-HSA-2395516	Electron transport from NADPH to Ferredoxin	0.028356422
R-HSA-8949275	RUNX3 Regulates Immune Response and Cell Migration	0.028356422
R-HSA-977606	Regulation of Complement cascade	0.030496245
R-HSA-2046106	alpha-linolenic acid (ALA) metabolism	0.030866342
R-HSA-3322077	Glycogen synthesis	0.031048097
R-HSA-5652084	Fructose metabolism	0.034127685
R-HSA-947581	Molybdenum cofactor biosynthesis	0.034127685
R-HSA-2855086	Ficolins bind to repetitive carbohydrate structures on the target cell surface	0.039482963
R-HSA-916853	Degradation of GABA	0.039482963
R-HSA-5423646	Aflatoxin activation and detoxification	0.040106927
R-HSA-189445	Metabolism of porphyrins	0.043294687
R-HSA-211999	CYP2E1 reactions	0.044274267
R-HSA-8963889	Assembly of active LPL and LIPC lipase complexes	0.044274267
R-HSA-9006931	Signaling by Nuclear Receptors	0.046346114

Supplementary Table S2. The Reactome pathway analysis of negatively correlated genes with three SOX genes using SARC-TCGA transcriptome datasets.

Pathway identifier	Pathway name	Entities p-Value
R-HSA-8863678	Neurodegenerative Diseases	3.21E-04
R-HSA-8862803	Deregulated CDK5 triggers multiple neurodegenerative pathways in Alzheimer's disease models	3.21E-04
R-HSA-9645723	Diseases of programmed cell death	3.65E-04
R-HSA-9615017	FOXO-mediated transcription of oxidative stress, metabolic and neuronal genes	8.49E-04
R-HSA-168256	Immune System	0.001064552
R-HSA-936837	Ion transport by P-type ATPases	0.001762662
R-HSA-8950505	Gene and protein expression by JAK-STAT signaling after Interleukin-12 stimulation	0.001861506
R-HSA-9020591	Interleukin-12 signaling	0.002451274
R-HSA-447115	Interleukin-12 family signaling	0.003182541
R-HSA-1280215	Cytokine Signaling in Immune system	0.0039805
R-HSA-9614085	FOXO-mediated transcription	0.004149368
R-HSA-6798695	Neutrophil degranulation	0.007825043
R-HSA-909733	Interferon alpha/beta signaling	0.011422393
R-HSA-9020558	Interleukin-2 signaling	0.012332974
R-HSA-983712	Ion channel transport	0.013999915
R-HSA-351200	Interconversion of polyamines	0.01408332
R-HSA-391160	Signal regulatory protein family interactions	0.015830803
R-HSA-449147	Signaling by Interleukins	0.01700624
R-HSA-1614517	Sulfide oxidation to sulfate	0.019317195
R-HSA-877300	Interferon gamma signaling	0.020288412
R-HSA-451927	Interleukin-2 family signaling	0.040850135
R-HSA-1614558	Degradation of cysteine and homocysteine	0.04595268
R-HSA-913531	Interferon Signaling	0.046636296

Supplementary Table S3. Accession numbers of the SOX protein sequences (NCBI).

Gene symbol	Protein Accession Number
SOX1	EAX09158.1
SOX2	EAW78354.1
SOX3	AAF73059.1
SOX4	EAW55429.1
SOX5	P35711.3
SOX6	P35712.3
SOX7	Q9BT81.1
SOX8	P57073.1
SOX9	P48436.1
SOX10	P56693.1
SOX11	P35716.2
SOX12	O15370.2
SOX13	Q9UN79.3
SOX14	O95416.1
SOX15	O60248.1
SOX17	Q9H6I2.1
SOX18	P35713.2
SOX21	Q9Y651.1
SOX30	O94993.1
SRY	AFG33955.1
